# New Dengue Virus Type 1 Genotype in Colombo, Sri Lanka

**DOI:** 10.3201/eid1711.101893

**Published:** 2011-11

**Authors:** Hasitha A. Tissera, Eng Eong Ooi, Duane J. Gubler, Ying Tan, Barathy Logendra, Wahala M.P.B. Wahala, Aravinda M. de Silva, M.R. Nihal Abeysinghe, Paba Palihawadana, Sunethra Gunasena, Clarence C. Tam, Ananda Amarasinghe, G. William Letson, Harold S. Margolis, Aruna Dharshan De Silva

**Affiliations:** Ministry of Health, Colombo, Sri Lanka (H.A. Tissera, M.R.N. Abeysinghe, P. Palihawadana); London School of Hygiene and Tropical Medicine, London, UK (H.A. Tissera, C.C. Tam); Duke–National University of Singapore Graduate Medical School, Singapore (E.E. Ooi, D.J. Gubler, Y. Tan); Genetech Research Institute, Colombo (B. Logendra, A.D. De Silva); University of North Carolina School of Medicine, Chapel Hill, North Carolina, USA (W.M.P.B. Wahala, A.M. de Silva); Medical Research Institute, Colombo (S. Gunasena); International Vaccine Institute, Seoul, South Korea (A. Amerasinghe, G.W. Letson, H.S. Margolis)

**Keywords:** dengue, dengue hemorrhagic fever, dengue virus, viruses, genotype, flavivirus, molecular epidemiology, phylogeny, surveillance, Sri Lanka

## Abstract

The number of cases and severity of disease associated with dengue infection in Sri Lanka has been increasing since 1989, when the first epidemic of dengue hemorrhagic fever was recorded. We identified a new dengue virus 1 strain circulating in Sri Lanka that coincided with the 2009 dengue epidemic.

Dengue virus (DENV) is a flavivirus transmitted by *Aedes* spp. mosquitoes. There are 4 distinct DENV serotypes (DENV-1–4). Infection with a single serotype leads to long-term protective immunity against the homologous serotype but not against other serotypes (*1*). Globally, dengue is an emerging disease that causes an estimated 50–100 million infections, 500,000 dengue hemorrhagic fever (DHF) cases, and 22,000 deaths annually (*2**,**3*).

Epidemiologic and other studies indicate that risk factors for severe dengue include secondary infection with a heterologous serotype, the strain of infecting virus, and age and genetic background of the host. Studies are under way to further explore the role of these factors in severe disease (*1**,**4*).

In Sri Lanka, serologically confirmed dengue was first reported in 1962 (*5*), but although all 4 virus serotypes were present and there were cases of DHF, only since 1989 has DHF been considered endemic to Sri Lanka (*5*). Dengue was made a reportable disease in Sri Lanka in 1996, and the largest epidemic (35,008 reported cases, 170 cases/100,000 population, and 346 deaths) occurred in 2009 (*6*). DHF epidemics in 1989 and 2002–2004 were associated with emergence of new clades of DENV-3 (*7**,**8*). We report a new DENV-1 genotype introduced to Sri Lanka before the 2009 epidemic.

## The Study

The study was approved by the Ethical Review Committee of the Faculty of Medicine, University of Colombo, Sri Lanka, and the Institutional Research Board of the International Vaccine Institute, Seoul, South Korea. Serum samples were obtained in 2009 and early 2010 from patients as part of a Pediatric Dengue Vaccine Initiative (PDVI) fever surveillance study in Colombo, Sri Lanka. Samples were originally tested for dengue by reverse transcription PCR at Genetech Research Institute (Colombo, Sri Lanka). A random subset of dengue-positive samples of all 4 serotypes was sent to the Program in Emerging Infectious Diseases Laboratory at Duke–National University of Singapore Graduate Medical School, Singapore, for virus isolation and sequencing.

RNA was extracted from virus isolates, subjected to standard reverse transcription PCR to confirm the presence of dengue virus, and serotyped as described (*7*). Samples processed at Duke–National University of Singapore underwent whole-genome sequencing as described (*9*). Using DENV-1 isolates from Sri Lanka obtained from dengue cases in 1983, 1984, 1997, 2003, and 2004 (*7*) and representative DENV-1 sequences for the 4 genotypes, we constructed a phylogenetic tree by using MEGA5 software (*10*) (Figure; Table).

**Figure:**
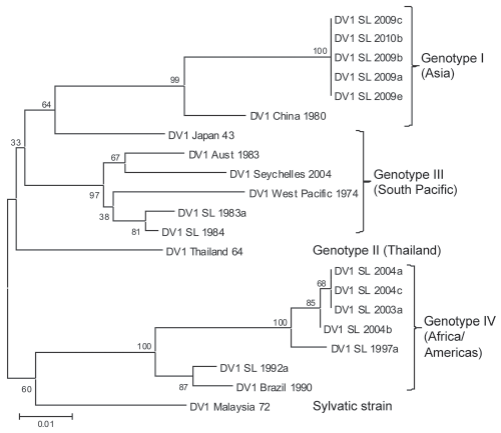
Phylogenetic tree of dengue virus 1 (DENV-1) serotype viruses from Sri Lanka (SL), 2009–2010, and other DENV-1 viruses. The tree is based on a 498-bp (nt 2056–2554) fragment that encodes portions of the envelope protein and nonstructural protein 1. Phylogenetic analysis was conducted by using MEGA5 (*10*). Percentages of replicate trees in which the associated taxa clustered in the bootstrap test (1,000 replicates) are shown next to the branches. Genotype I (Asia) includes SL isolates from 2009–2010; genotype III (South Pacific) includes SL isolates from the early 1980s; genotype IV (Africa/Americas) includes SL isolates from the 1990s and early 2000s. Classification and naming of DENV-1 genotypes are based on the report by Rico-Hesse (*11*). DV, dengue virus. Scale bar indicates nucleotide substitutions per site.

**Table:** Dengue virus type 1 strains used in analysis of new dengue virus genotype, Colombo, Sri Lanka

Virus strain*	Location	Subtype	Year isolated	GenBank accession no.
DV1_Aust_1983	Australia	III	1983	AB074761
DV1_West_Pacific_1974	Western Pacific	III	1974	U88535
DV1_Brazil_1990	Brazil	IV	1990	AF226685
DV1_China_1980	People’s Republic of China	I	1980	AF350498
DV1_Japan_43	Japan	I	1943	AB074760
DV1_Malaysia_72	Malaysia	Sylvatic	1972	EF457905
DV1_Seychelles_2004	Seychelles	III	2004	DQ285561
DV1_SL_1983a	Sri Lanka	III	1983	FJ225443
DV1_SL_1984	Sri Lanka	III	1984	FJ225444
DV1_SL_1992a	Sri Lanka	IV	1992	FJ225445
DV1_SL_1997a	Sri Lanka	IV	1997	FJ225446
DV1_SL_2003a	Sri Lanka	IV	2003	FJ225447
DV1_SL_2004a	Sri Lanka	IV	2004	FJ225448
DV1_SL_2004b	Sri Lanka	IV	2004	FJ225449
DV1_SL_2004c	Sri Lanka	IV	2004	FJ225450
DV1_Thailand_64	Thailand	II	1964	AF180818
DV1_SL_2009a	Sri Lanka	I	2009	HQ891313
DV1_SL_2009b	Sri Lanka	I	2009	HQ891314
DV1_SL_2009c	Sri Lanka	I	2009	HQ891315
DV1_SL_2009e	Sri Lanka	I	2009	JN054256
DV1_SL_2010b	Sri Lanka	I	2010	JN054255

*Strains are indicated as genotype_location_year.

The 4 DENV serotypes found in Sri Lanka have been classified into genotypes according to the nomenclature described by Rico-Hesse (*11*). The earliest isolates found in 1983 and 1984 belong to South Pacific genotype III. More recent isolates obtained during surveillance efforts during 1997–2004 belong to Africa/America genotype IV, indicating that at some point between the early 1980s and the mid 1990s, there was a DENV-1 genotype shift. Analysis of viruses isolated in 2009 indicated that another Asia genotype I of DENV-1 has been introduced into Sri Lanka (Figure) (*7*). This Asia genotype I virus appears to be responsible for the 2009 epidemic of dengue fever and DHF.

## Conclusions

A feature of the epidemiology of dengue in Sri Lanka was the lack of DHF in the early 1980s and the increase in the number of severe dengue cases since 1989, more so after 2000. This finding was observed despite seroprevalence rates remaining largely the same over time as reported in a previous study (*12*) and in the current PDVI study (*13*).

Previous epidemics (1989 and 2002–2004) showed a correlation with evolution of DENV-3 genotype III in Sri Lanka, where emergence of new clades of DENV-3 genotype 3 showed a correlation with large increases in the number of reported cases and the geographic range of the virus (*7**,**8*). A similar observation was reported for Puerto Rico by Bennett et al., who compared data for DEN2 and DEN4 over 20 years and found that dominant clades were replaced by viral subpopulations existing within the population (*14*) and in the South Pacific region for DENV-2, where a similar clade replacement occurred (*15*). These clade changes were accompanied by positive selection in the nonstructural protein 2A (NS-2A) gene for DENV-4 and the envelope, premembrane, NS-2A, and NS-4A genes for DENV-2.

Our results indicate that introduction of a new DENV-1 genotype coincided with the 2009 dengue epidemic in Sri Lanka. Studies are underway to determine if the proportion of DENV-1 cases in 2009 was greater than in previous years and to assess the role of this new DENV-1 genotype in the severe epidemic of 2009. Further studies are needed to determine if this new genotype has spread to other countries in the region.
